# Supplementary material for: The microbiome biomarkers of pregnant women’s vaginal area predict preterm prelabor rupture in Western China
Source: Front Cell Infect Microbiol. 2024 Oct 31;14:1471027. doi: 10.3389/fcimb.2024.1471027 (PMC11560878; doi:10.3389/fcimb.2024.1471027)
Supplement: Supplementary file 1 [file DataSheet1.zip › compare_1/Community/Pieplot/family/sample/C23.family.pieplot.pdf]

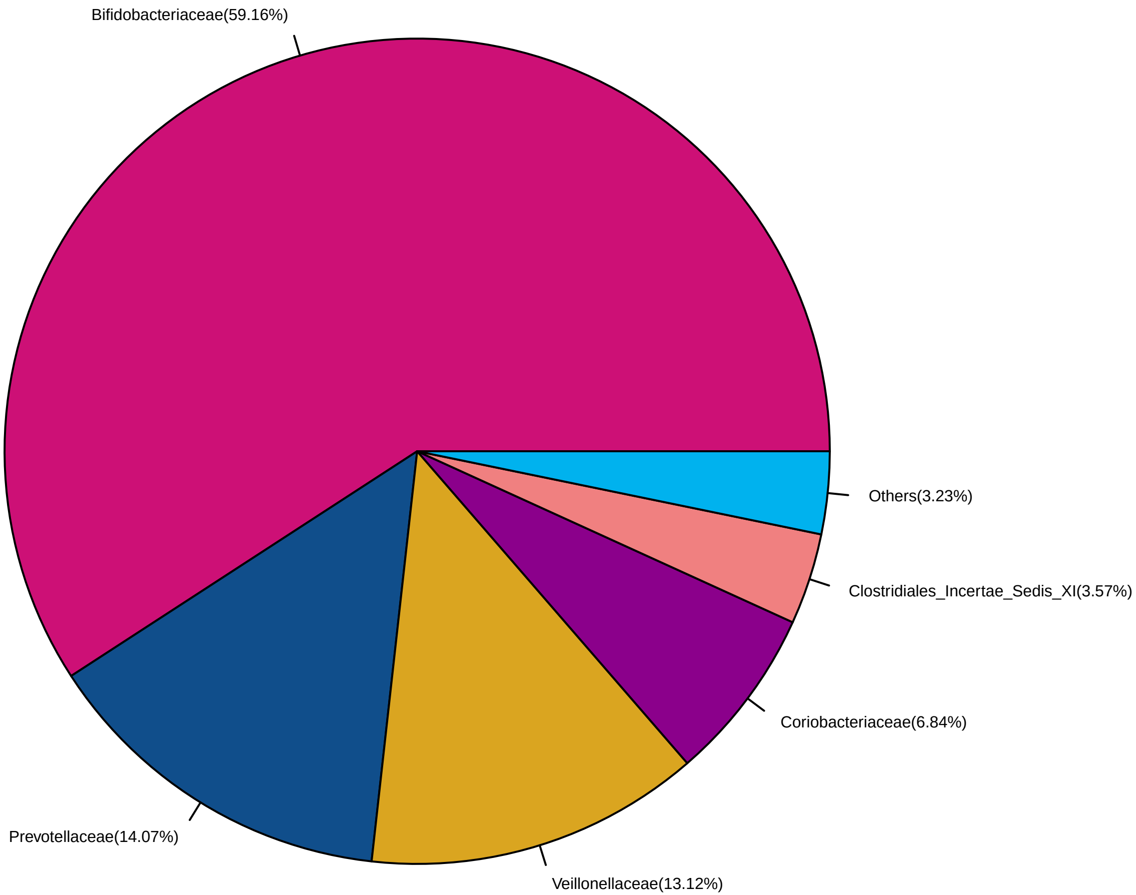
Bifidobacteriaceae(59.16%)
Others(3.23%)
Clostridiales_Incertae_Sedis_XI(3.57%)
Coriobacteriaceae(6.84%)
Veillonellaceae(13.12%)
Prevotellaceae(14.07%)
